# Use of walking modifications, perceived walking difficulty and changes in outdoor mobility among community-dwelling older people during COVID-19 restrictions

**DOI:** 10.1007/s40520-021-01956-2

**Published:** 2021-08-20

**Authors:** Heidi Leppä, Laura Karavirta, Timo Rantalainen, Merja Rantakokko, Sini Siltanen, Erja Portegijs, Taina Rantanen

**Affiliations:** 1 Gerontology Research Center and Faculty of Sport and Health Sciences, University of Jyväskylä, Jyväskylä, Finland; 2 JAMK University of Applied Sciences, Institute of Rehabilitation, Jyväskylä, Finland; 3 Finnish Institute for Health and Welfare, Helsinki, Finland

**Keywords:** Aging, Compensation, Mobility, Participation, Social isolation, SARS-CoV-2

## Abstract

**Background:**

Outdoor mobility enables participation in essential out-of-home activities in old age.

**Aim:**

To compare changes in different aspects of outdoor mobility during COVID-19 restrictions versus two years before according to self-reported walking.

**Methods:**

Community-dwelling participants of AGNES study (2017–2018, initial age 75–85) responded to AGNES-COVID-19 postal survey in spring 2020 (*N* = 809). Life-space mobility, autonomy in participation outdoors, and self-reported physical activity were assessed at both time points and differences according to self-reported walking modifications and difficulty vs. intact walking at baseline were analyzed.

**Results:**

Life-space mobility and autonomy in participation outdoors had declined (mean changes -11.4, SD 21.3; and 6.7, SD 5.3, respectively), whereas physical activity had increased (5.5 min/day, SD 25.1) at follow-up. Participants perceiving walking difficulty reported the poorest baseline outdoor mobility, a steeper decline in life-space mobility (*p* = 0.001), a smaller increase in physical activity (*p* < 0.001), and a smaller decline in autonomy in participation outdoors (*p* = 0.017) than those with intact walking. Those with walking modifications also reported lower baseline life-space mobility and physical activity, a steeper decline in life-space mobility and a smaller increase in physical activity those with intact walking (*p* < 0.001 for both).

**Discussion:**

Participants reporting walking modifications remained the intermediate group in outdoor mobility over time, whereas those with walking difficulty showed the steepest decline in outdoor mobility and hence potential risk for accelerated further functional decline.

**Conclusion:**

Interventions should target older people perceiving walking difficulty, as they may be at the risk for becoming homebound when environmental facilitators for outdoor mobility are removed.

## Introduction

Outdoor mobility indicates an individual’s actual mobility behavior and perceived possibilities for participation in essential out-of-home activities [1, 2]. The concept includes all types of journeys outside home, whether on foot or by other means of transportation, and thus requires some level of walking ability [2]. During the aging process, age-related diseases and functional decline may increase the risk for walking difficulty [3], in turn hindering possibilities to participate in out-of-home activities and leading to further decline in outdoor mobility [4]. However, before perceiving actual walking difficulty, older people noticing the first signs of functional decline may seek to maintain their outdoor mobility by modifying their walking behavior, for example, using an aid or walking more slowly [4].

During spring 2020, multiple actions were taken globally to slow down the spread of the SARS-CoV-2 virus responsible for COVID-19, especially among high-risk populations. In Finland, the government announced a state of emergency and, as a general guideline, advised older people to limit their physical contacts and avoid crowded areas. Restaurants, libraries, and indoor sport facilities were closed, and many cultural and civic society events and organized classes were canceled. Particular concerns were expressed regarding the potentially adverse consequences of these restrictions on older people’s outdoor mobility and physical activity, as older people typically accumulate most of their physical activity while running daily errands, attending various events or making social visits [5–7].

Thus far, studies evaluating the effects of the COVID-19 restrictions and lockdowns have focused on changes in one aspect of outdoor mobility at a time among older people and have mostly utilized cross-sectional data based on convenience samples [8–11]. In these studies, the majority of older people reported a decrease in their physical activity during the COVID-19 restrictions [8–10]. Lower scores for life-space mobility, referring to individuals’ actual mobility behavior in daily life, and for active aging were observed in our previous study comparing data collected during the COVID-19 restrictions with data collected two years earlier [11]. In our previous prospective study [4] conducted prior to the COVID-19 pandemic, perceived walking difficulty preceded the decline in life-space mobility. However, the use of walking modifications enabled older people to postpone the decline in life-space mobility and in autonomy in participation outdoors [12]. It is thus possible that the COVID-19 restrictions have had different effects on older people’s life-space mobility, autonomy in participation outdoors and physical activity, according to their use of walking modifications or perceived walking difficulty. We hypothesized that older people who perceived walking difficulty prior to the COVID-19 pandemic would show a steeper decline in various aspects of their outdoor mobility during the COVID-19 restrictions compared to those with intact walking.

The first aim of this study was to examine levels and changes in life-space mobility, autonomy in participation outdoors, and self-reported physical activity among older people during the COVID-19 restrictions compared to two years earlier. The second aim was to investigate whether the levels and changes in these various aspects of outdoor mobility differed between those reporting intact walking, walking modifications, or difficulty in walking a 2-km (km) distance at baseline.

## Methods

### Study design and participants

This study presents longitudinal results of the ‘Active Aging – resilience and external support as modifiers of the disablement outcome’ (AGNES) observational cohort study. Follow-up data (AGNES-COVID-19) were collected via postal questionnaires during the COVID-19 restrictions (May and June 2020) and these data were compared to baseline data (collected 2017–2018). The study protocol of the AGNES study [13] and non-respondent analyses of both datasets have been reported previously [11, 14]. Briefly, the AGNES study is an observational study of three birth cohorts (aged 75, 80 and 85 years). A random sample based on age and residence in specific postal code areas in Jyväskylä (Finland) was drawn from the Digital and Population Data Services Agency in Finland. Inclusion criteria were being resident in the study area, community-dwelling, willing to participate, able to communicate, and provide an informed consent. At baseline, structured personal interview was conducted in participants homes (N = 1 018). At follow-up, a postal questionnaire was sent to the 985 baseline participants not known to have died or been transferred to an institutional care facility, and who had not withdrawn their consent [11]. Altogether, 809 responses were received. Seven participants had difficulty answering the questionnaire or preferred an interview and were thus interviewed over the phone. During collecting the follow-up data, the number of confirmed COVID-19 cases was low in the study area (102 cases, population 253 000, 21 municipalities) [11].

### Measurements

*Self-reported walking modifications and difficulty in 2-km* were assessed at baseline [15, 16]. First, perceived difficulty in walking a distance of 2-km was asked with the question: “*Do you have difficulty in walking 2-km?”* The response alternatives varied from “able to manage without difficulty” to “unable to manage even with help”. Second, those using walking modifications at baseline were identified by asking those who reported being able to walk without difficulty an additional question: “*Have you noticed any of the following changes due to your health or physical functioning when walking 2-km?*” The response options were walking slower, taking rest breaks during walking, using an aid, having reduced the frequency of walking, and having given up walking distances of 2-km (“yes” or “no”). For the analyses, participants were categorized as follows: 1) *intact walking* (no difficulty nor modifications, reference), 2) *walking modifications* (no difficulty and at least one modification), and 3) *walking difficulty* (at least some difficulty).

Life-space mobility, autonomy in participation outdoors and self-reported physical activity were measured at baseline and during the COVID-19 restrictions. *Life-space mobility* was measured with the Finnish version of the University of Alabama at Birmingham Study of Aging Life-Space Assessment [17, 18]. The Life-Space Mobility Assessment is a validated measure designed to capture individuals’ actual mobility behavior in daily life. Participants were asked on how many days per week during the four weeks preceding the assessment they reached each life-space level, and if they needed help from other people or assistive devices. A higher life-space composite score indicates greater life-space mobility (range 0–120) [17].

*Autonomy in participation outdoors* was measured using the respective subscale of the Impact on Participation and Autonomy Questionnaire (IPA) [19]. The IPA is a validated measure for assessing participation and autonomy in clinical populations and older people and can be used as a whole questionnaire or as subscales [19, 20]. The autonomy outdoors subscale comprises five items assessing a person’s satisfaction with his/her possibilities to take part in activities outside the home: visiting relatives and friends, making trips and traveling, spending leisure time, meeting other people, and living life the way one wants to. Each item is scored from 0 (very good possibilities) to 4 (very poor possibilities). A higher sum score indicates more restrictions in autonomy in participation outdoors (range 0–20).

*Self-reported physical activity* was assessed using the Yale Physical Activity Survey for older adults [21]. Participants were asked how many times they had performed vigorous physical activity and leisure walking for at least 10 min during the past month and the usual duration of these sessions. Total minutes per day were calculated using the following formula [14]: (frequency*duration)/7. Finally, mean daily vigorous physical activity and leisure walking minutes were summed.

*Age and sex* were obtained from the Finnish National Population Register at the sampling stage. In addition, information on years of education, number of chronic conditions, depressive symptoms, and lower extremity function were collected at baseline during structured home interviews by trained interviewers and used only for descriptive purposes. *Years of education,* as an indicator of socioeconomic status, was self-reported. *Number of chronic conditions* was calculated as the sum of individual chronic conditions from a list of physician-diagnosed chronic conditions followed by an open-ended question on any other chronic diseases the participant might have [13]. *Depressive symptoms* were assessed with the Center for Epidemiologic Studies Depression Scale, CES-D (range 0–60, with higher scores indicating more depressive symptoms) [22]. The Short Physical Performance Battery, SPPB (range 0–12, with higher scores indicating better lower extremity function) including balance, walking speed and chair stands were used to assess *lower extremity function* [23].

### Statistical analyses

Baseline characteristics were compared between the self-reported walking categories using cross-tabulation with chi-square test for categorical variables and one-way ANOVA with a Bonferroni test (post hoc comparisons) for normally distributed continuous variables. Overall longitudinal changes in life-space mobility and autonomy in participation outdoors scores, and in physical activity minutes were calculated using paired samples *t-*test. Generalized Estimation Equations (GEE) linear models [24] with an unstructured working correlation matrix were used to compare changes in life-space mobility, autonomy in participation outdoors and self-reported physical activity over the follow-up between the self-reported walking categories. We estimated main effects (group difference) and time interaction effects (group by time). Adjusting the models for age and sex did not change the main and time interaction effects, and thus only age- and sex-adjusted models are reported. The models were adjusted only for age and sex, because the purpose was to study changes over time at the individual level in life-space mobility, autonomy in participation outdoors and self-reported physical activity related to the COVID-19 restrictions according participants’ self-reported walking at baseline.

This study comprised AGNES participants who also participated in the AGNES-COVID-19 survey (*N* = 809). Age and sex were available for all participants, whereas information on self-reported walking was missing for 12 participants; hence, the final models comprised 797 participants. Missing autonomy in participation outdoors scores was imputed for follow-up participants with only one missing item (*n* = 14) using the mean of the available items. In addition, in the GEE models, multivariate imputation by chained equations was used to calculate scores for missing baseline and follow-up total scores for life-space mobility (baseline *n* = 4, follow-up *n* = 6), autonomy in participation outdoors (baseline *n* = 13, follow-up *n* = 27) and minutes for self-reported physical activity (baseline *n* = 14, follow-up *n* = 16). Including participants with imputed items, total scores or, minutes did not change the results based on the sensitivity analyses (data not shown). IBM SPSS version 24 for Windows (IBM Corp., Armonk, NY) was used for statistical analyses. The results were regarded as statistically significant if the p value was < 0.05.

## Results

Baseline characteristics according to self-reported walking are shown in Table 1. Based on the post hoc comparisons, those reporting walking difficulty (*n* = 268) were older, less educated and had more chronic conditions, depressive symptoms, and poorer lower extremity function than those reporting intact walking (*n* = 396) (*p* < 0.008 for all). Participants with walking modifications (*n* = 133) did not differ from those with intact walking in years of education (*p* = 0.097) or number of chronic conditions (*p* = 0.139). They formed an intermediate group in their lower extremity function and depressive symptoms scores between those with intact walking and those with walking difficulty (*p* < 0.015 for all) and had fewer chronic conditions than those with walking difficulty (*p* < 0.001).

**Table 1:** Participants’ Background Characteristics by Self-Reported Ability to Walk 2-km at Baseline (*n* = 797)

Characteristics	Intact Walking (*n* = 396)	Modifications (*n* = 133)	Difficulty (*n* = 268)	
	Mean (SD)	Mean (SD)	Mean (SD)	*P* value
Age, years	77.7 (3.2)	78.8 (3.6)	79.7 (3.7)	< 0.001 ^a^
Education, years	12.3 (4.3)	11.3 (4.3)	11.2 (4.2)	0.005 ^a^
SPPB, score	11.0 (1.2)	10.2 (1.7)	8.8 (2.7)	< 0.001 ^a^
CES-D, score	6.6 (5.9)	8.5 (7.2)	10.7 (7.7)	< 0.001 ^a^
No. of chronic diseases	2.8 (1.7)	3.1 (1.8)	4.4 (2.2)	< 0.001 ^a^
Life-space mobility, score	79.8 (14.9)	73.5 (14.9)	61.4 (19.7)	< 0.001 ^a^
Autonomy in participation outdoors, score	4.0 (3.2)	5.4 (3.3)	6.6 (4.0)	< 0.001 ^a^
Self-reported physical activity, minutes	43.3 (20.4)	35.0 (18.1)	24.1 (16.9)	< 0.001 ^a^
Women, % (n)	52.3 (207)	57.1 (76)	68.3 (183)	< 0.001 ^b^

*SD* Standard Deviation, *CES-D* Center for Epidemiologic Studies Depression Scale, *SPPB* Short Physical Performance Battery

^a^ Tested with one-way analysis of variance

^b^ Tested with chi-square test

**Fig. 1:**
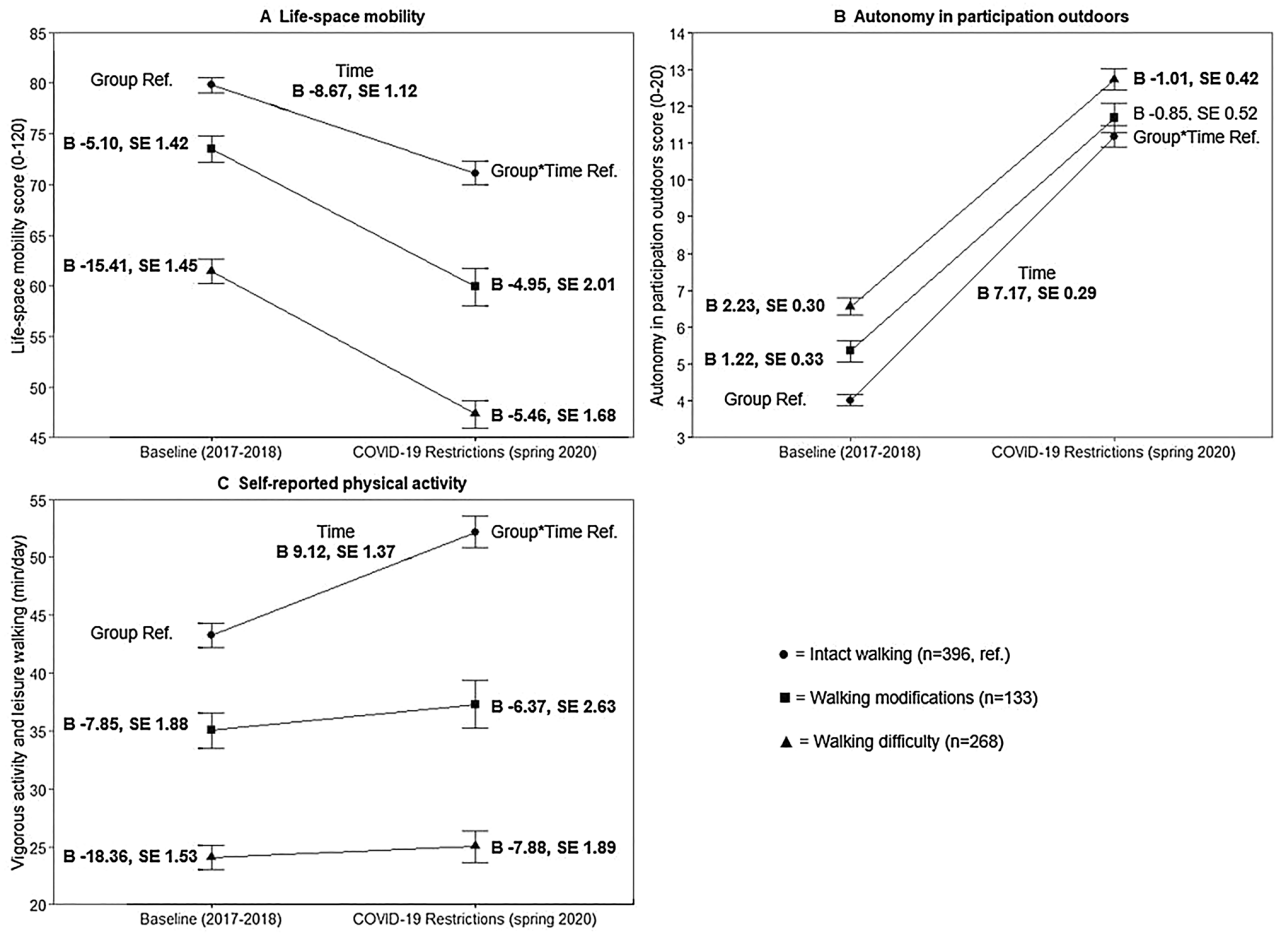
Differences at baseline and in changes over time in (**A**) life-space mobility (higher scores indicate greater life-space mobility), (**B**) autonomy in participation outdoors (higher scores indicate more restrictions in autonomy) and (**C**) self-reported physical activity, vigorous activity, and leisure walking minutes (with standard error) according to self-reported walking at baseline. GEE models are adjusted for age and sex

Life-space mobility scores decreased on average  − 11.4 points (SD 21.3) in all participants during the COVID-19 restrictions when compared to their scores two years before (72.6, SD 18.6 vs. 61.2, SD 24.7). Those with walking difficulty had a lower life-space mobility score at baseline (Table 1 and Fig. 1) and showed a steeper decline over time than those with intact walking. Those with walking modifications also had a lower life-space mobility score at baseline and showed a steeper decline over the follow-up than those with intact walking.

Participants were less satisfied with their possibilities to participate in activities outside their homes than two years earlier (5.1, SD 3.7 vs. 11.7, SD 5.1), as their autonomy in participation outdoors scores increased on average by 6.7 (SD 5.4) points over the follow-up. While those with walking difficulty reported poorer opportunities to participate in out-of-home activities than those with intact walking at baseline (Table 1 and Fig. 1), the decrease in autonomy in participation outdoors at follow-up was greater among those reporting intact walking at baseline. In turn, while those with walking modifications perceived worse autonomy in participation outdoors at baseline than those reporting intact walking, the change at follow-up in these two groups was similar.

Daily time spent in vigorous physical activities and in leisure walking had increased on average by 5.3 (SD 25.0) minutes among all participants at follow-up during the COVID-19 restrictions (35.3, SD 20.8 vs. 40.6, SD 27.5). At baseline, those reporting walking difficulty or use of walking modifications accumulated less daily vigorous physical activity and walking minutes than those with intact walking (Table 1 and Fig. 1). Among those with intact walking, daily vigorous physical activity and walking minutes had increased from baseline to the COVID-19 restrictions, whereas it remained more stable among those with walking difficulty and those with walking modifications.

## Discussion

The present findings indicate that while life-space mobility and autonomy in participation outdoors declined, physical activity increased among community-dwelling older people between the pre-COVID baseline and the follow-up two years later during the COVID-19 restrictions. People with intact walking in 2-km distance had the most favorable baseline scores for life-space mobility, autonomy in participation outdoors and physical activity. Moreover, although their life-space mobility and autonomy in participation outdoors declined, the amount of time spent in vigorous physical activity and walking increased. In turn, those reporting walking difficulty showed a more unfavorable level of outdoor mobility at baseline and the steepest decline in life-space mobility at follow-up during the COVID-19 restrictions. The participants with walking modifications remained in an intermediate position in all three outcome variables at both measurement points.

The decline in life-space mobility during the COVID-19 restrictions compared to two years before the pandemic was clinically meaningful [17] and notably steeper (on average 6–18 points) than in our previous study (on average 1–5 points) with a similar cohort and follow-up period [12]. Reduced life-space mobility may have a significant influence on older persons’ everyday lives, as it is associated with multiple adverse health outcomes, such as increased risk for further functional decline [25], nursing home admission [26] and mortality [27]. Older people with walking difficulty and those with walking modifications showed the steepest decline in life-space mobility and were at the highest risk for restricted life-space mobility (from 61 to 47 points, 74 to 60, respectively) during the COVID-19 restrictions, meaning that they rarely moved outside of their immediate neighborhood [18]. The observed change in life-space mobility among those using walking modifications suggests that the compensatory effect of using walking modifications decreased during COVID-19 restrictions. Thus, in the present study, instead of postponing the decline in outdoor mobility [4], the use of walking modifications was an indicator of preclinical disability and a further reduction in walking activity [28]. Walking difficulty often coexists with cognitive impairments [29] and fear of moving outdoors [30], which may also compromise participation in everyday activities and accelerate the decline in life-space mobility [31]. Older people with walking difficulty may have been and may continue to be at heightened risk of becoming homebound during the COVID-19 restrictions especially if the restrictions on outdoor mobility are prolonged and effective interventions are not offered. Being homebound is a serious situation, as it is associated with a high mortality rate [32] and dependency in self-care [33].

Autonomy in participation outdoors indicates an individual’s level of satisfaction with their opportunities to move outdoors and for instance to leave the home to visit relatives and friends as often as one wants [19]. Avoiding seeing other people was strongly recommended in Finland during the COVID-19 restrictions, and thus it is only logical that participants’ perceived autonomy in participation outdoors declined (average change 6–7 points). In contrast, in our previous study, conducted during a period with no restrictions in place, perceived autonomy in participation outdoors remained almost unchanged (average change 0–1 points) with a cohort and follow-up time comparable to those in the present study [12]. Hence, it is likely that the observed changes in participants’ autonomy in participation outdoors reflect the impact of the COVID-19 restrictions rather than the impacts of a person’s individual ability [19]. Our observation that people with intact walking perceived a steepest decline in their autonomy in participation outdoors compared to those perceiving walking difficulty further supports this explanation. Autonomy is an essential goal of rehabilitation as it reflects participants’ own perceptions of their possibilities to live life as they want to [19] and contributes to maintaining life satisfaction [34]. Therefore, although no cut-point for a meaningful change in the autonomy in participation outdoors score has been established, the observed seven-point mean decline may have had a meaningful negative effect on the participants’ lives. However, how this decline in autonomy in participation outdoors, if prolonged, affects older people’s lives warrants further research.

Older people’s physical activity increased in the present study, whereas in previous studies conducted in Italy and Spain it decreased during the COVID-19 restrictions [8–10]. This unexpected inconsistency between findings may be explained by the different strategies used to prevent the spread of the virus. Italy and Spain were in nationwide lockdowns and their citizens were not allowed to leave their homes without a valid reason [35, 36], whereas in Finland, no curfew was imposed at any time during spring 2020. In addition, we assessed physical activity as time spent in vigorous activity and leisure walking. In addition to exercising outdoors, vigorous activity may have included at-home activities, such as indoor cycling or strength-training. Overall, our findings suggest, in line with a previous study [37], that older people with intact walking compensated, at least partly, for their lost participation in social activities by exercising at home or walking for leisure during the COVID-19 restrictions. In contrast, the lowest levels of physical activity were observed, as in previous study [38] among older people perceiving walking difficulty. Therefore, interventions aiming to increase physical activity should especially target people perceiving walking difficulty or using walking modifications.

The study has its limitations. Owing to the COVID-19 restrictions, the follow-up data were collected using postal questionnaires. Thus, we cannot be sure who responded to the questionnaire or whether some participants misunderstood some of the questions. In addition, physical activity was self-reported, which may have led to overestimation of physical activity levels. We cannot rule out the possibility that changes in health are affecting the associations found. However, considering the greater changes in outdoor mobility in the present study compared to an earlier cohort [12] and the low rates of markedly worsened health during the follow-up, we consider that effects of the COVID-19 restrictions likely to be of greater magnitude. Overall, the effects of these limitations to the results are likely to be small.

The strengths of this study include the large population-based sample of community-dwelling older people and the longitudinal study design with data collected prior to and during the COVID-19 restrictions. In addition, our study contributes further knowledge on the consequences of the COVID-19 restrictions: first, by assessing differences based on 2-km walking categories, second, by assessing three important aspects of older people’s outdoor mobility, and third, by comparing the results over time. Previous studies, in contrast, have focused solely on changes in physical activity [8–10] or used a cross-sectional design and targeted self-selected convenience samples [8, 10]. Finally, the present study opens the way for future research.

## Conclusion

Older people with intact walking coped better with the COVID-19-related restrictions than those with walking modifications or difficulty, as they were able to compensate for suspended social activities by increasing their physical activity. In future, special attention should be paid to older people perceiving walking difficulty, as they seem to be at the highest risk for becoming homebound when environmental facilitators to outdoor mobility are removed. When comparing our findings to previous study, with a similar cohort and living environment, we noticed that the decline in life-space mobility and autonomy in participation during the first wave of COVID-19 exceeded the decline that would naturally have occurred due to the aging process over a 2-year period. As this study describes the situation in the early phase of the pandemic, further studies are needed to investigate the effects of prolonged COVID-19 restrictions on older people’s outdoor mobility. In addition, studies should examine how experiencing restricted life-space mobility and autonomy in participation outdoors during the first wave of COVID-19 affects older people’s subsequent walking ability, and whether older peoples’ life-space mobility and autonomy in participation outdoors returns to pre-COVID levels after the COVID-19 pandemic restrictions have been lifted.
